# Rapid Measurement of Erythrocyte Sedimentation Rate Using a Tube Positioned at a 45° Angle Compared With the Westergren Reference Method

**DOI:** 10.1155/bmri/8860256

**Published:** 2026-06-19

**Authors:** Francis Agyei Amponsah, Benedicta Serwaa Obeng, Otchere Addai-Mensah, Lilian Antwi Boateng, Benedict Sackey, Isaac Acheampong, Prince Adoba, Edward Yaw Afriyie, Anthony Dwamena, Samuel Kwasi Appiah, Philip Amo-Kodieh, Felicia Broni Nartey

**Affiliations:** ^1^ Department of Medical Diagnostics, Faculty of Allied Health Sciences, Kwame Nkrumah University of Science and Technology, Kumasi, Ghana, knust.edu.gh; ^2^ Department of Haematology, Komfo Anokye Teaching Hospital, Kumasi, Ghana, kathhsp.org; ^3^ Sunyani Municipal Hospital, Sunyani, Ghana; ^4^ College of Health, Yamfo, Ghana; ^5^ Department of Haematology, School of Allied Health Sciences, University for Development Studies, Tamale, Ghana, uds.edu.gh; ^6^ Laboratory Department, St. John of God Hospital, Duayaw Nkwanta, Ghana

**Keywords:** blood donors, erythrocyte sedimentation rate, pregnant women, Westergren method

## Abstract

**Background:**

The erythrocyte sedimentation rate (ESR) is a routine hematological test that can detect and track an increase in the body′s inflammatory activity caused by infections, tumors, or autoimmune diseases. Although the test has lower specificity than C‐reactive protein in assessing inflammation, it is widely used in resource‐limited countries such as Ghana due to its low cost and ease of performance. The conventional Westergren method of ESR estimation takes 1 h, and this could hinder the availability of results in routine clinical workflows. It is in view of this that the present study sought to reduce the turnaround time without compromising on test accuracy by using a method that involved positioning the tube at an angle of 45°.

**Methods:**

This experimental analytical method comparison study recruited 100 participants (50 pregnant and 50 healthy blood donors) aged between 15 and 40 years. Five milliliters of venous blood was taken from each participant and added to trisodium citrate anticoagulant in a fixed blood‐to‐anticoagulant ratio of 4:1. For each study participant, two Westergren–Katz tubes were filled with the diluted blood to the 200 mm mark, and one was held vertically at 90° while the other was set at an angle of 45° at room temperature. The ESR value was recorded after 1 h for the vertically (90°) set tube, while the ESR value was read every minute for 10 min for the tube placed at an angle of 45°.

**Results:**

ESR results obtained from the 6^th^ to the 10^th^ minutes for the tubes placed at an angle of 45° showed comparable results to those recorded at the end of an hour for the tubes set vertically at 90° in both pregnant women and the healthy blood donors (*p* > 0.05). In both study groups, Bland–Altman plots revealed that ESR measurements taken at the 8^th^ minute at 45° had the best agreement with minimal bias to ESR values at 90° for 1 h.

**Conclusion:**

This study proved that the turnaround time for the ESR test could be reduced to 8 min if the tube is positioned at an angle of 45°.

## 1. Introduction

The erythrocyte sedimentation rate (ESR) or length of sedimentation reaction in blood (LSRB) test is a routine hematological test done in clinical laboratories and measures how quickly blood cells sediment in a test tube in 1 h [1, 2]. The test detects and tracks increases in the body′s inflammatory activity resulting from infections, tumors, or autoimmune diseases [2–4]. Thus, inflammation is characterized by higher ESR due to an increase in levels of acute phase reactants such as fibrinogen, immunoglobulins, and other plasma proteins, which facilitate the formation of larger red blood cell (RBC) aggregates [5, 6].

The Westergren method is the gold standard for determining the ESR [7]. The test involves placing anticoagulated whole blood into a Westergren tube, which is positioned upright, and recording the fall of the RBC column in an hour. In healthy conditions, factors that facilitate red cell aggregation and rouleaux formation are balanced by the opposing effects of albumin and surface charges of the red cells. However, in inflammatory conditions, the effect of the red cell charges and albumin is subdued by acute phase proteins from the liver and by immunoglobulins from B‐lymphocytes: These promote RBC aggregation and rouleaux formation, thus increasing RBC sedimentation [8]. Even though the ESR is not an emergency diagnostic test, a shorter testing time could enhance clinical decision‐making and clinical workflows [9]. More so, a shorter testing time will enable laboratory personnel to perform other duties.

ESR is affected by factors including plasma fibrinogen and globulin levels, rouleaux formation, erythrocyte size and shape, and mechanical and technical concerns [10]. An increased fibrinogen concentration increases the ESR value [11]. ESR decreases gradually when kept over 6 h at room temperature [12]. A tilted ESR tube setup and increased vibrations can also increase the ESR value [4], and an angle of three degrees from vertical can increase the ESR by 30% [3]; hence, there is an effect of the degree of tube inclination on the ESR. A tilted ESR tube positioned at an angle below 90° results in RBC aggregation along the lower side, while plasma rises along the upper side, causing a decrease in the time at which RBCs sediment in the ESR tube, thereby reducing ESR testing time [13].

Although ESR is a nonspecific and less sensitive marker of inflammation compared to C‐reactive protein (CRP), it is an important test used to evaluate inflammation in certain disease conditions and monitor treatment in others, such as Hodgkin′s lymphoma. The conventional ESR test continues to be used in clinical practice in a number of developing countries because it is cheaper, does not use electricity or expensive equipment/instrumentation, consumables are readily available, and can easily be performed in remote clinics and health centers [14, 15]. Although the analytical time for the conventional test is 1 h, this could be unduly prolonged due to inadequate laboratory staffing, batching, and reporting processes. In resource‐limited settings such as Ghana, ESR is the main test used to assess inflammatory conditions. However, to improve the efficiency of laboratory workflows, patient flows, and clinical decision‐making, it would be beneficial to find alternative means of conducting the test without increasing its cost while reducing the turnaround time. Thus, we compared the conventional method in which the tubes were set at an angle of 90° for 1 h to a modified ESR test, whereby tubes were positioned at an angle of 45°, and the rate of fall was read at every minute for 10 min to determine the ideal time at which the ESR value of the modified method statistically compared reliably with the standard ESR method.

## 2. Materials and Methods

### 2.1. Study Design/Setting

This experimental analytical method comparison study was conducted from the 1^st^ to the 20^th^ of July 2024 at Asuofua Health Centre in Kumasi, Ashanti Region of Ghana. The study compared ESR values obtained from the standard Westergren method with those obtained using a modified technique involving positioning the tube at an angle of 45°.

Asuofua is a suburb of Kumasi, the capital city of the region. Kumasi has a population of about 1,722,806 people and lies within the plateau of the southwest physical region, which ranges between 250 and 300 m above sea level.

The Asuofua Health Centre is one of the primary health care centers in Kumasi, with a bed capacity of 70. The health center has five wards and provides general health services, laboratory diagnostic services, and reproductive health services, among others.

### 2.2. Participant Recruitment

A simple random sampling technique was adopted in recruiting 100 participants, comprising 50 apparently healthy blood donors and 50 pregnant women who visited the health facility. The sample size was deemed adequate for this preliminary exploratory analytical comparison study aimed at assessing the agreement between the modified ESR technique and the standard Westergren reference method across participants with relatively low and elevated ESR values.

### 2.3. Inclusion and Exclusion Criteria

Participants aged between 15 and 40 years who were apparently healthy blood donors and pregnant women attending antenatal care services were recruited into the study. Blood donors comprised males and females who had successfully passed routine predonation screening protocols.

### 2.4. Ethical Consideration

Approval was obtained from the Committee on Human Research Publication and Ethics (CHRPE) of the School of Medicine and Dentistry at the Kwame Nkrumah University of Science and Technology, Kumasi (CHRPE/AP/521/24). Written informed consent was obtained from participants and guardians of participants younger than 18 years.

### 2.5. Sample Collection and Laboratory Investigations

A volume of 5 mL of venous blood was aseptically taken from each participant into trisodium citrate anticoagulant at a fixed blood‐to‐anticoagulant ratio of 4:1. The samples were gently inverted 10 times immediately after collection to ensure proper mixing of blood with anticoagulant to prevent clotting. All tests were performed within 30 min of sample collection to minimize preanalytical variation. For each study participant, two standardized Westergren–Katz tubes (Evancare Medical Co. Ltd., China) were filled with the diluted blood to the 200 mm mark. One ESR tube was positioned at an angle of 90° (vertically) in accordance with the standard Westergren ESR protocol, while the other was positioned at an angle of 45° and kept at room temperature (23°C–25°C) on a stable vibration‐free laboratory bench to minimize technical variability. The positions of the tubes were achieved by using a commercially available ESR stand, which has predetermined angle slots set at three different inclinations (90°, 60°, and 45°). The present study used the 90° and 45° angles of tube positions. The ESR values were recorded after 1 h for the conventional method, while the ESR values were recorded every minute for 10 min for the modified technique, which is the tube positioned at an angle of 45°. For each measurement, the distances from the zero graduation mark to the upper level of the sedimented RBC column were recorded.

## 3. Statistical Analysis

Data collected during the research was subsequently transferred into Microsoft Excel and analyzed using GraphPad Prism (version) and IBM SPSS 27.0. The data distribution was assessed and analyzed with nonparametric tests. The Mann–Whitney test was used to compare data between the pregnant women and blood donors. Friedman′s test was used to compare ESR between the different time points at 45° and for 1 h at 90°. A Bland–Altman plot was used to evaluate the agreement between the new method at various time points at 45° (nonsignificant from 90° for 1 h) and the established method at 90° for 1 h within each group. For all comparisons, a *p* value less than 0.05 was considered statistically significant.

## 4. Results

### 4.1. Differences in ESR Values Between Healthy Blood Donors and Pregnant Women

Table 1 compared the ESR values between the healthy blood donors and the pregnant women at 90° for 1 h and at 45° for 1–10 min. With the exception of ESR values for tubes positioned at an angle of 45° for 1 min, the ESR values in the healthy blood donors were consistently lower than those of the pregnant women at all corresponding angles of position of tubes and time intervals. The differences were statistically significant (*p* < 0.001).

**Table 1:** Comparing corresponding ESR values between donors and pregnant women at 90° for 1 h and at 45° for 1–10 min.

ESR	Donors (*N* = 50) Median (Q_1_–Q_3_)	Pregnant women (*N* = 50) Median (Q_1_–Q_3_)	*p* value
90°/1 h (mm/h)	15 (8.75–24.25)	39 (25.75–60)	**< 0.001**
45°/1 min (mm/h)	0 (0–0)	0 (0–0)	> 0.999
45°/2 min (mm/h)	0 (0–0)	1 (1–2.25)	**< 0.001**
45°/3 min (mm/h)	1 (1–5)	5 (2–11.25)	**< 0.001**
45°/4 min (mm/h)	3 (1–7.25)	15 (8–23.25)	**< 0.001**
45°/5 min (mm/h)	6 (3–12)	19 (11.75–34)	**< 0.001**
45°/6 min (mm/h)	9 (4–18.5)	25 (19.75–42)	**< 0.001**
45°/7 min (mm/h)	12.5 (5–22.25)	30 (23.75–50)	**< 0.001**
45°/8 min (mm/h)	16.5 (8–26.25)	38.5 (25–58)	**< 0.001**
45°/9 min (mm/h)	18 (10–29.75)	41.50 (28.75–61.75)	**< 0.001**
45°/10 min (mm/h)	20.5 (12.75–35.25)	46.5 (32.5–66.25)	**< 0.001**

*Note:* The Mann–Whitney test was used for the analysis; *N* is the number of participants; data are presented as medians with 25^th^ (Q_1_) and 75^th^ (Q_3_) interquartile ranges in parentheses. Differences in ESR values obtained when the tube is tilted at 45° at different time points (1–10 min) versus ESR values obtained when the tube is positioned vertically for 1 h (60 min) for each of the respective groups (healthy blood donors and pregnant women). Bold *p* values indicate significant differences.

Abbreviation: mm/h, millimeters per hour.

Table 2 presents the comparison between ESR values at 90° for 1 h and ESR values at 45° obtained at different time points (1–10 min) among healthy blood donors (controls) and pregnant women (cases). In both pregnant women and healthy blood donors, there were no significant differences between the results obtained at the 6^th^–10^th^ minutes at an angle of 45° and the results obtained for the tubes positioned at an angle of 90° for an hour. However, there were significant differences in the results obtained at the 1^st^–5^th^ minutes at the angle of 45° (*p* < 0.05).

**Table 2:** ESR values at 90° for 1 h against values at 45° for 1–10 min.

ESR	Blood donors (*N* = 50) Median (Q_1_–Q_3_)	Pregnant women (*N* = 50) Median (Q_1_–Q_3_)
90°/1 h (mm/h)	15 (8.75–24.25)	39 (25.75–60)
45°/1 min (mm/h)	0 (0–0) ^∗^	0 (0–0) ^∗^
45°/2 min (mm/h)	0 (0–0) ^∗^	1 (1–2.25) ^∗^
45°/3 min (mm/h)	1 (1–5) ^∗^	5 (2–11.25) ^∗^
45°/4 min (mm/h)	3 (1–7.25) ^∗^	15 (8–23.25) ^∗^
45°/5 min (mm/h)	6 (3–12) ^∗^	19 (11.75–34) ^∗^
45°/6 min (mm/h)	9 (4–18.5)	25 (19.75–42)
45°/7 min (mm/h)	12.5 (5–22.25)	30 (23.75–50)
45°/8 min (mm/h)	16.5 (8–26.25)	38.5 (25–58)
45°/9 min (mm/h)	18 (10–29.75)	41.50 (28.75–61.75)
45°/10 min (mm/h)	20.5 (12.75–35.25)	46.5 (32.5–66.25)
*p* value	*p* < 0.001	*p* < 0.001

*Note:* Friedman ^′^s test was used for the analysis; *N* is the number of participants; data are presented as medians with 25^th^ (Q_1_) and 75^th^ (Q_3_) interquartile ranges in parentheses.

Abbreviation: mm/h, millimeters per hour.

∗ means the data is significant when compared to the measured ESR value at 90° for 1 h.

**Figure 1:**
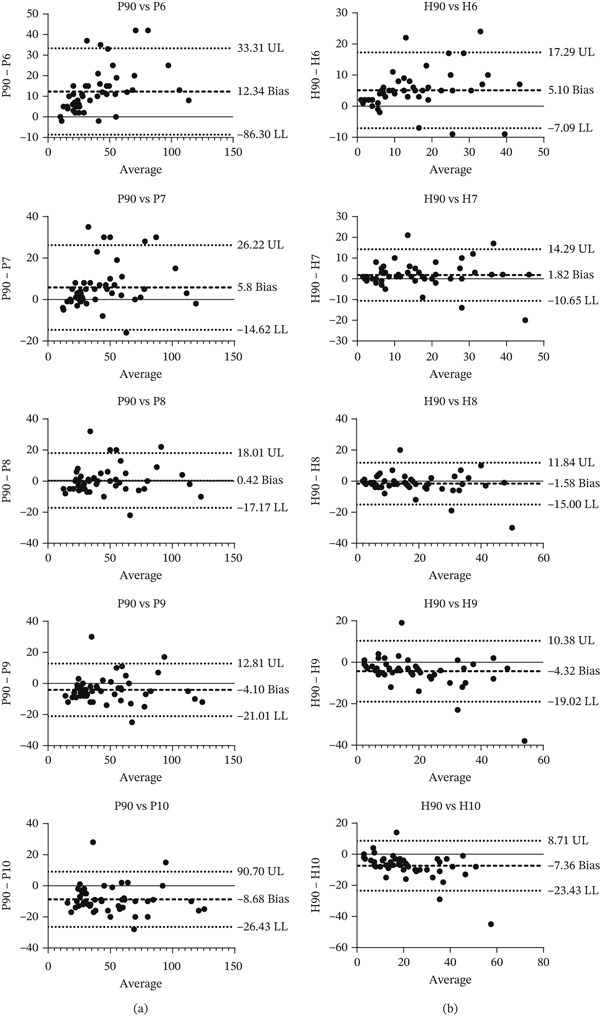
Bland–Altman plots of ESR values of the tube positioned at an angle of 45° for every minute up to the tenth minute compared to those of the tube positioned at an angle of 90° for 1 h. (a) The Bland–Altman plot in pregnant women. (b) The Bland–Altman plot in healthy donors. P90 are ESR values measured at 90° for 1 h in pregnant women; H90 are ESR values measured at 90° for 1 h in healthy donors; P6, P7, P8, P9, and P10 are ESR values measured at 45° for 6, 7, 8, 9, and 10 min in pregnant women, respectively; and H6, H7, H8, H9, and H10 are ESR values measured at 45° for 6, 7, 8, 9, and 10 min in healthy donors, respectively. UL is the upper limit of agreement; LL is the lower limit of agreement.

In Figure 1, Bland–Altman plots were used to compare the 6^th^–10^th^ minute measurements at 45° in each group with the standard 90° for the 1‐h method for both cases and controls to know which minute is in close agreement with the 90° for the 1‐h measurements. The ESR values obtained at the 45° angle for 8 min had the best agreement with minimal bias to the standard 90° for 1 h in both the pregnant women and the healthy blood donors.

## 5. Discussion

The ESR is a nonspecific inflammatory indicator that measures the rate at which RBCs sediment. ESR is a widely used hematological test for monitoring responses to therapy and identifying inflammatory conditions such as rheumatoid arthritis, infections, temporal arteritis, and polymyalgia rheumatica [16]. It is measured in millimeters by the distance between the meniscus and the upper layer of the sedimented RBCs in citrated blood in a given period, which is usually 1 h [17]. Although there are automated systems that have reduced this long turnaround time, these are not popular in resource‐limited settings such as Ghana due to how expensive these automated systems are. Thus, it is not surprising that the Westergren reference method is the widely used option in most developing countries, such as Ghana, in assessing inflammation in the body. This study therefore evaluated a modified ESR technique involving a tube positioned at an angle of 45° to determine whether a reliable result could be obtained within a shorter time.

ESR values for pregnant women are higher than those of healthy blood donors [13] and corroborate findings from the present study, whereby, for each method, ESR values were higher in pregnant women compared to the apparently healthy blood donors. Physiological changes during pregnancy, such as increased fibrinogen levels, plasma volume expansion, and decreased red cell count/hemoglobin concentration [18], enhance rouleaux formation and increase erythrocyte sedimentation. A decrease in plasma viscosity (low hematocrit) as a result of fluid retention results in higher ESR values in pregnancy.

If the ESR tube is tilted at an angle below 90°, RBCs aggregate along the lower side while plasma rises along the upper side, causing a decrease in the time at which the RBCs sediment in the tube, and this in turn reduces the testing time for ESR [13]. This was confirmed by the present study, in that positioning the ESR tube at an angle of 45° resulted in increasing ESR values, which reached a maximum at the 10^th^ minute. Moreover, the participants′ ESR values obtained within the first 5 min for the tubes positioned at an angle of 45° differed significantly from their respective ESR values obtained when their duplicate tubes were held vertically for 60 min and thus were not reliable when compared to the reference method.

However, there were no statistically significant differences between the results obtained from the sixth minute through to the tenth minute. Thus, ESR values obtained from these time points are comparable to those obtained when the tube is held vertically for 60 min, but there was a need to find the precise minute that had the value closest to the value obtained for the conventional method. The rapid increases in ESR values within the first 5 min, which plateaued after the sixth minute, could be attributed to the rapid and initial rouleaux formation by the negatively charged erythrocytes. The process of rouleaux formation is enhanced by plasma proteins such as immunoglobulins and fibrinogen, which reduce intererythrocytic repulsions [4]. Rouleaux formation increases the mass of these erythrocyte aggregates, which facilitates faster sedimentation. On the other hand, the majority of erythrocytes have fully aggregated after the 6^th^ minute, and the rate of sedimentation plateaus with a slower sedimentation of single cells. Hematocrit, plasma viscosity, and RBC morphology influence the overall rate of sedimentation, but the initial rapid phase is a reflection of rouleaux formation and sedimentation dynamics.

Hence, a Bland–Altman plot was utilized to evaluate the agreement between the new method at various time points at 45° (nonsignificant from 90° for 1 h) and the established method at 90° for 1 h within each group. The comparison aimed to identify the time point at 45° that most closely agreed with the standard method. Bland–Altman plots comparing the 6^th^–10^th^ minute measurements at 45° in each group with the standard 90° for the 1‐h method showed that the measurements taken at the 8^th^ minute when the tubes were positioned at an angle of 45° in both cases and controls were in close agreement with the values obtained when their duplicate tubes were positioned at an angle of 90° for 1 h. In the conventional ESR setup at 90°, red cells undergo sedimentation through three phases. An initial aggregation phase where no sedimentation occurs is followed by a subsequent constant rate of falling and a final phase of red cell packing. Despite the variability of duration of the various phases due to variability of red cell morphology, disease state, immunological response, and relative proportion of various blood components, the result of this experiment shows that a modified method of ESR setup with the tube positioned at an angle of 45° gives an accurate result that is comparable to the standard Westergren method when the test is read at 8 min. Previous findings by [19] found that positioning the tube at an angle of 45° for 10.5–11.5 min produced values that correlated significantly with the standard method, and this supports the reduction in turnaround time but differs in the optimal timing. These discrepancies may be attributed to temperature variations between study sites since higher ambient temperatures found in Ghana (study site of the present work) can reduce the time it takes for RBCs to sediment compared to cooler climates like in Sri Lanka, where the previous study was conducted.

Recent studies evaluating rapid ESR technologies and modified Westergren methods support the concept that ESR turnaround time could be drastically reduced without compromising on analytical agreement. Although most of these studies used automated instrumentation and achieved reliable results when compared to the Westergren method [2, 4, 16], the present study provides a low‐cost alternative ideal for clinical laboratories with limited resources.

This study has several limitations: A relatively small sample size was used, which restricted the assessment of extreme ESR values. Additionally, the present study did not collect participants′ demographics and detailed clinical data, as the aim was to compare the analytical performance of two methods, but not a true patient‐level clinical study. Also, we were unable to assess the possible influence of hematocrit, red cell morphology (e.g., ESR is lower in sickle cell individuals at steady state [absence of crisis] since the red cell shape prevents rouleaux formation but is increased during crises [20]), protein concentrations [10], type of tube, and operator variability on the ESR values. Therefore, further validation by considering these factors is necessary before clinical implementation by respective laboratories. Nonetheless, the findings offer a promising approach to significantly reduce ESR turnaround time without compromising diagnostic accuracy or reliability, especially in West Africa.

## 6. Conclusion

This study established that the angle position of the ESR tube has an effect on the ESR values. Positioning the ESR tube at an angle of 45° produced ESR values comparable to those obtained using the conventional method when measurements were recorded at the 8^th^ minute. The new technique, therefore, offers a simple, rapid, and cost‐effective approach for reducing turnaround time without compromising analytical reliability. Thus, this new technique, when adopted, will improve the efficiency of clinical decision‐making and patient flows, particularly in resource‐limited settings such as Ghana.

## Author Contributions

Conceptualization: Francis Agyei Amponsah, Benedicta Serwaa Obeng, Otchere Addai‐Mensah, Lilian Antwi‐Boateng, Benedict Sackey, Edward Yaw Afriyie, Isaac Acheampong, Prince Adoba, and Philip Amo‐Kodieh. Data curation: Benedicta Serwaa Obeng, Prince Adoba, Felicia Broni Nartey, Philip Amo‐Kodieh, Anthony Dwamena, and Samuel Kwasi Appiah. Formal analysis: Francis Agyei Amponsah, Prince Adoba, Samuel Kwasi Appiah, Benedicta Serwaa Obeng, and Isaac Acheampong. Investigation: Francis Agyei Amponsah, Benedicta Serwaa Obeng, Anthony Dwamena, and Felicia Broni Nartey. Methodology: Francis Agyei Amponsah, Otchere Addai‐Mensah, Edward Yaw Afriyie, Prince Adoba, and Benedict Sackey. Project Administration: Francis Agyei Amponsah, Lilian Antwi‐Boateng, Philip Amo‐Kodieh, and Anthony Dwamena. Resources: Francis Agyei Amponsah, Benedicta Serwaa Obeng, Samuel Kwasi Appiah, Isaac Acheampong, Edward Yaw Afriyie, Otchere Addai‐Mensah, Lilian Antwi‐Boateng, and Benedict Sackey. Validation: Francis Agyei Amponsah, Otchere Addai‐Mensah, Benedict Sackey, and Samuel Kwasi Appiah. Writing—original draft: Francis Agyei Amponsah, Benedicta Serwaa, Otchere Addai‐Mensah, Lilian Antwi‐Boateng, Benedict Sackey, Edward Yaw Afriyie, Isaac Acheampong, Prince Adoba, Philip Amo‐Kodieh, Felicia Broni Nartey, Samuel Kwasi Appiah, and Anthony Dwamena. Writing—review and editing: Francis Agyei Amponsah, Benedicta Serwaa, Otchere Addai‐Mensah, Lilian Antwi‐Boateng, Benedict Sackey, Edward Yaw Afriyie, Isaac Acheampong, Prince Adoba, Philip Amo‐Kodieh, Felicia Broni Nartey, Samuel Kwasi Appiah, and Anthony Dwamena.

## Funding

No funding was received for this manuscript.

## Ethics Statement

Ethical approval was obtained from the Committee on Human Research Publication and Ethics (CHRPE) of the School of Medicine and Dentistry at the Kwame Nkrumah University of Science and Technology, Kumasi (CHRPE/AP/521/24). Permission was obtained from managers of the Asuofua Health Centre in Kumasi, Ashanti Region of Ghana. Written informed consent was obtained from participants and guardians of participants younger than 18 years.

## Conflicts of Interest

The authors declare no conflicts of interest.

## Data Availability

All relevant data are within the article.
